# Spo0A Differentially Regulates Toxin Production in Evolutionarily Diverse Strains of *Clostridium difficile*

**DOI:** 10.1371/journal.pone.0079666

**Published:** 2013-11-13

**Authors:** Kate E. Mackin, Glen P. Carter, Pauline Howarth, Julian I. Rood, Dena Lyras

**Affiliations:** Department of Microbiology, Monash University, Clayton, Victoria, Australia; Institute Pasteur, France

## Abstract

*Clostridium difficile* is an important pathogen of humans and animals, representing a significant global healthcare problem. The last decade has seen the emergence of epidemic BI/NAP1/027 and ribotype 078 isolates, associated with the onset of more severe disease and higher rates of morbidity and mortality. However, little is known about these isolates at the molecular level, partly due to difficulties in the genetic manipulation of these strains. Here we report the development of an optimised Tn*916*-mediated plasmid transfer system, and the use of this system to construct and complement *spo0A* mutants in a number of different *C. difficile* strain backgrounds. Spo0A is a global regulator known to control sporulation, but may also be involved in the regulation of potential virulence factors and other phenotypes. Recent studies have failed to elucidate the role of Spo0A in toxin A and toxin B production by *C. difficile*, with conflicting data published to date. In this study, we aimed to clarify the role of Spo0A in production of the major toxins by *C. difficile*. Through the construction and complementation of *spo0A* mutants in two ribotype 027 isolates, we demonstrate that Spo0A acts as a negative regulator of toxin A and toxin B production in this strain background. In addition, *spo0A* was disrupted and subsequently complemented in strain 630Δ*erm* and, for the first time, in a ribotype 078 isolate, JGS6133. In contrast to the ribotype 027 strains, Spo0A does not appear to regulate toxin production in strain 630Δ*erm*. In strain JGS6133, Spo0A appears to negatively regulate toxin production during early stationary phase, but has little effect on toxin expression during late stationary phase. These data suggest that Spo0A may differentially regulate toxin production in phylogenetically distinct *C. difficile* strain types. In addition, Spo0A may be involved in regulating some aspects of *C. difficile* motility.

## Introduction

*Clostridium difficile* is the causative agent of a range of intestinal diseases. In humans, *C. difficile* infection (CDI) can present as diarrhoea, which may range in severity from mild to severe [1]; in some cases CDI may progress to toxic megacolon, perforation of the bowel, sepsis and death. *C. difficile* is also an emerging infection in animals, with disease appearing particularly common in neonatal pigs and horses, however, it may occur sporadically in other species [2]. The spectrum of CDI in animals is similar to that in humans, ranging from asymptomatic colonisation to fulminant disease [3]. Disease is caused by the production of two major toxins, toxin A and toxin B. Both toxins are monoglucosyltransferases that inactivate host cell Rho-family GTPases, resulting in the disruption of the host cell actin cytoskeleton and cell death [4], [5]. This leads to a loss of tight junction integrity within the gut, which facilitates the movement of fluid into the intestinal lumen, followed by the onset of diarrhoea [4]. The infectious particle is the spore; for this reason, germination and sporulation are also important in the disease process. Once in the host gut, spore germination occurs and the vegetative cell expresses factors required for survival and pathogenesis. *C. difficile* then sporulates again, and is excreted from the host due to the diarrhoea which is symptomatic of this infection [6], [7].

The master regulator of sporulation is Spo0A, which controls the transition of the bacterium into the spore form. Spo0A may also be associated with the regulation of putative virulence factors and other phenotypes such as motility [8]–[11]. Recent work has examined the role of Spo0A in the regulation of toxin A and toxin B production by *C. difficile* [12]–[14], however, the findings from these studies have not been consistent. Underwood *et al*. (2009) have suggested that Spo0A positively regulates toxin A and toxin B expression, demonstrating a decrease in levels of toxin production in a 630Δ*erm spo0A* mutant [13]. However, Rosenbusch *et al*. (2012) have more recently demonstrated that disruption of *spo0A* in 630Δ*erm* does not affect toxin production levels [12]. Unfortunately, neither study complemented the mutation of *spo0A* nor tested independent mutants, making it impossible to assess the potential role of secondary mutations in the phenotypes of these strains. Deakin *et al*. (2012) recently reported that a R20291 *spo0A* mutant caused more severe disease in a murine model than the wild type strain. They associated this increase in severity with an increase in the amount of toxin A and toxin B produced by the mutant *in vitro*, which was reduced (albeit not to wild type levels) upon subsequent complementation with the wild-type gene [14], suggesting that Spo0A may negatively regulate toxin A and toxin B production in strain R20291.

*C. difficile* represents a significant global healthcare problem. This problem has escalated in the last decade following the emergence of so-called “hypervirulent” or epidemic BI/NAP1/027 *C. difficile* strains [15], [16] such as R20291. Associated with an increase in both the morbidity and mortality attributable to CDI [15], [17], these variants are currently spreading throughout the developed world. More recently, the emergence of a second group of *C. difficile* strains, classified as ribotype 078, has been reported [18]. These isolates have also been associated with more severe disease, particularly in comparatively younger people and in community-associated cases of disease [18]–[20]. Ribotype 078 strains are also often associated with animal disease, particularly in calves and neonatal pigs [21]. Phylogenetic analysis has shown that this strain type is highly divergent from other types, which may reflect the different host niches in which these isolates are often found [22], [23]. However, little is known about these strains at the molecular level, and to date there have been no reports documenting the genetic manipulation of ribotype 078 strains.

The genetic manipulation of *C. difficile* has in the past proved difficult and until recently many strains remained genetically refractory. We recently developed techniques that utilised the transfer machinery of the conjugative transposon Tn*916* to facilitate the efficient transfer of plasmids into previously refractory strains of *C. difficile* [24]. Here we report the further optimisation of this technique, specifically through the use of a *Bacillus subtilis* donor strain which facilitates more efficient plasmid transfer into the clostridia, particularly *C. difficile*. This method was used to construct and complement *spo0A* mutants in two ribotype 027 strains, in the ribotype 012 derivative 630Δ*erm* and in the ribotype 078 porcine isolate JGS6133. Using these strains, we sought to clarify the role of Spo0A in the regulation of toxin A and toxin B production by *C.difficile* in ribotype 027 isolates and in 630Δ*erm*, as well as investigate if Spo0A was involved in toxin regulation in a ribotype 078 isolate. We demonstrate that Spo0A negatively regulates toxin A and toxin B production in the ribotype 027 strains M7404 and R20291. In the ribotype 078 strain JGS6133, Spo0A appears to negatively regulate toxin A and toxin B production during early stationary phase but not at later stages of growth. By contrast, Spo0A does not appear to be involved in the regulation of toxin production in630Δ*erm* at all. These data suggest that the regulation of toxin production by Spo0A may have adapted specifically in various *C. difficile* strain types. The influence of Spo0A upon motility or growth on a solid surface also appears to vary in different strain backgrounds, further highlighting the heterogeneity of *C. difficile.*

## Materials and Methods

### Bacterial strains and growth conditions

The characteristics and origins of all recombinant strains and plasmids are shown in Tables 1 and 2. All bacteriological culture media were obtained from Oxoid, except BBL Agar Grade A from BD, used in the motility experiments. All clostridial strains were cultured in heart infusion supplemented with yeast extract, L-cysteine and glucose (HIS) or TY medium [25] unless otherwise stated, in an atmosphere of 10% H_2_, 10% CO_2_, and 80% N_2_ at 37°C in a Don Whitley A300 anaerobic work station. *B. subtilis* and *Escherichia coli* were cultured in 2×YT medium aerobically at 37°C, with shaking for broth cultures. All antibiotics were purchased from Sigma-Aldrich and were used at the following concentrations: D-cycloserine (250 µg/ml), cefoxitin (8 µg/ml), thiamphenicol (10 µg/ml), tetracycline (10 µg/ml), chloramphenicol (25 µg/ml for *E. coli*, 30 µg/ml for the clostridia or 5 µg/ml for *B. subtilis*), erythromycin (10 µg/ml), and lincomycin (20 µg/ml).

**Table 1:** Bacterial strains used in this study.

Strain	Characteristics	Source/Reference
*B. subtilis*		
BS34A	*B. subtilis* strain CU2189 derivative with 1 chromosomal copy of Tn*916*	[33]
DLL2003	BS34A (Tn*916*, pDLL1)	This study
DLL2004	BS34A (Tn*916*, pDLL4)	This study
DLL2005	BS34A (Tn*916*, pDLL16)	This study
*E. coli*		
TOP10	F- *mcr*A Δ(*mrr-hsd*RMS-*mcr*BC) φ80*lac*ZΔM15 Δ*lac*X74 *nup*G *rec*A1 *ara*D139 Δ(*ara-leu*)7697 *gal*U *gal*K *rps*L(Str^R^) *end*A1 λ^-^	Invitrogen
*C. perfringens*		
JIR4225	*C. perfringens* strain JIR325 with 5 chromosomal copies of Tn*916*	[50]
JIR39	CW362 Chl^R^, Str^R^ (spontaneous)	[51]
SM101	Food poisoning isolate	[52]
*C. sordellii*		
ATCC9714	Type strain; TcsL^+^ TcsH^−^ Neu^+^ SDL^−^ PLC^+^	[53]
*C. septicum*		
BX96	Clinical Isolate; Csa^+^	[54]
*C. difficile*		
M7404	Canadian BI/NAP1/027 isolate	[55]
JIR8094	Erythromycin sensitive derivative of genome strain 630	[27]
VPI10463	PaLoc-positive *C. difficile* isolate	[56]
CD37	PaLoc-negative *C. difficile* isolate	[57]
CD196	French historic BI/NAP1/027 isolate	[58]
JGS6133	Porcine ribotype 078 isolate	[24]
R20291	UK BI/NAP1/027 isolate	[44]
630Δ*erm*	Erythromycin sensitive derivative of genome strain 630	[59]
DLL3001	M7404 (pDLL4)	[24]
DLL3011	M7404 *spo0A*::targetron	This study
DLL3034	M7404 *spo0A*::targetron (pDLL4)	This study
DLL3033	M7404 *spo0A*::targetron (pDLL16)	This study
DLL3100	R20291 (pDLL4)	This study
DLL3004	R20291 *spo0A*::targetron	This study
DLL3036	R20291 *spo0A*::targetron (pDLL4)	This study
DLL3051	R20291 *spo0A*::targetron (pDLL16)	This study
DLL3057	630▵*erm* (pDLL4)	This study
DLL3037	630▵*erm spo0A*::targetron	This study
DLL3048	630▵*erm spo0A*::targetron (pDLL4)	This study
DLL3047	630▵*erm spo0A*::targetron (pDLL16)	This study
DLL3058	JGS6133 (pDLL4)	This study
DLL3039	JGS6133 *spo0A*::targetron	This study
DLL3050	JGS6133 *spo0A*::targetron (pDLL4)	This study
DLL3049	JGS6133 *spo0A*::targetron (pDLL16)	This study

**Table 2:** Plasmids used in this study.

Plasmid	Characteristics	Source/Reference
pMTL007-*spo0A*	Clostridial targetron plasmid used for inactivation of *spo0A* in *C. difficile*, Tm^R^	[29]
pDLL4	*C. difficile* shuttle vector allows plasmid to be mobilised by Tn*916*, Tm^R^	[24]
pDLL1	pDLL4 (*Pst*I) pMTL007-*spo0A*(*Nsi*I; 6303 bp) (*C. difficile* targetron vector, mobilised by Tn*916*, used for inactivation of *spo0A*), Tm^R^	This study
pDLL16	pDLL4 (*Pst*I) DLP7/DLP8 PCR product (*Pst*I; 1204 bp) (*C. difficile spo0A* expression vector), Tm^R^	This study

### Molecular biology and PCR techniques

Plasmid DNA was isolated from *C. difficile*, *C. perfringens* and *B. subtilis* cultures using QIAprep spin miniprep columns (Qiagen). Cells were first lysed by incubating in buffer P1 (Qiagen) containing 30 mg/ml lysozyme at 37°C for 30 minutes, and plasmid DNA was subsequently isolated according to the manufacturer’s instructions. Genomic DNA was isolated from *C. difficile* using the modified method of Pospiech and Neumann (1995) [26] as outlined in O’Connor *et al.* (2006) [27]. Standard methods for the digestion, modification, ligation, and analysis of plasmid and genomic DNA were used [28]. Nucleotide sequence analysis was carried out using a PRISM BigDye Terminator cycle sequencing kit (Applied Biosystems) and detection was performed by Micromon at Monash University. Oligonucleotide primer sequences are listed in text. Unless otherwise stated, all PCR experiments were carried out with Phusion DNA polymerase (New England Biolabs) and the 2x Failsafe PCR buffer E (Epicentre) according to the manufacturer’s instructions.

### Construction of recombinant plasmids

For construction of the Tn*916*-transferable targetron plasmid, the *spo0A*-retargeted targetron element was excised from plasmid pMTL007-*spo0A* [29] using *Nsi*I and subcloned into the unique *Pst*I site of plasmid pDLL4 [24], resulting in the final construct pDLL1.

For construction of the *spo0A* complementation plasmid, PCR was performed using primers DLP7 (5′ AAACTGCAGGATAAAGGAAATTATAGAGATATGG 3′) and DLP8 (5′ AAACTGCAGCCAATGCCTTAATTAATTAAAAGCCTTAC 3′) to amplify the *spo0A* coding region and 300 bp upstream from chromosomal DNA extracted from *C. difficile* R20291. These primers also included *Pst*I sites to facilitate cloning of the PCR product into the base vector, pDLL4. The resulting 1204 bp PCR product was cloned into pCR-Blunt II-TOPO (Invitrogen), and then subcloned into the *Pst*I site of pDLL4, generating plasmid pDLL16.

### Transfer of plasmid DNA into *C. difficile* and other Clostridial spp. by conjugation

Recombinant plasmids were introduced into *B. subtilis* BS34A as described previously [30]. Conjugations using the resultant *B.subtilis* BS34A derivative as a donor strain were then performed as follows: A 20 ml HIS broth culture was inoculated with a 1 ml aliquot from an overnight *C. difficile* recipient strain culture and a 20 ml HIS broth culture was inoculated with a 1 ml aliquot from an overnight *B. subtilis* BS34A culture with appropriate selection. Both were then grown to mid-exponential phase. A 1 ml aliquot was removed from the *B. subtilis* culture and centrifuged to pellet the cells. The cell pellet was then resuspended in a 100 µl aliquot removed from the *C. difficile* culture. The cell suspension was then spread onto a HIS agar plate and incubated overnight at 37°C under anaerobic conditions. Bacterial growth was then harvested in sterile PBS before being spread onto HIS agar supplemented with thiamphenicol and incubated overnight as before. Following this incubation, bacterial growth was again harvested with PBS and dilutions subsequently spread onto HIS agar supplemented with D-cycloserine, cefoxitin and thiamphenicol as appropriate, and the plates incubated under anaerobic conditions for 24 to 72 h. Conjugations using a *C. perfringens* donor were performed as described previously [24].

### Isolation of *C. difficile spo0A* mutants

*C. difficile* transconjugants carrying pDLL1 were streaked onto HIS agar plates supplemented with thiamphenicol and incubated overnight under anaerobic conditions. Single colonies were then restreaked onto HIS agar supplemented with lincomycin for strains M7404 and R20291, or erythromycin for strains 630Δ*erm* and JGS6133, and incubated for 24 to 48 hours. All lincomycin or erythromycin resistant isolates were then cross-patched onto HIS agar supplemented with thiamphenicol to ensure loss of plasmid pDLL1 after insertion of the targetron into *spo0A*. Isolates resistant to erythromycin or lincomycin and sensitive to thiamphenicol were then analysed by PCR and Southern hybridisation analysis. All *C. difficile* transconjugants were also checked by tetracycline sensitivity, PCR or Southern blot, as appropriate, to ensure Tn*916* had not co-transferred with pDLL1 (data not shown).

### Complementation of *C. difficile spo0A* mutants

The *C. difficile spo0A* mutants were complemented by introducing pDLL16, which contains the wild type *spo0A* gene together with the 300 bp upstream region, using the same plasmid transfer method outlined above, with thiamphenicol used to select for transconjugants. Complementation was confirmed by plasmid rescue and phenotypic analysis. The vector pDLL4 was also introduced into the wild-type parental strains and mutant derivatives in order to maintain thiamphenicol selection across all strains during phenotypic analyses.

### Sporulation assays

HIS broth cultures were grown overnight and adjusted to an OD_600_ of 0.1 in pre-reduced HIS broth and incubated until reaching mid-exponential phase (OD_600_ between 0.4–0.6). This culture was then diluted 1 in 100 in fresh TY broth. After incubation for up to 72 hours, samples were plated onto HIS agar containing 1% (w/v) sodium taurocholate (New Zealand Pharmaceuticals) to determine total viable counts. Concurrently, a sample was heat-shocked at 65°C for 30 minutes, and then plated onto HIS agar containing sodium taurocholate to obtain heat-resistant viable counts. Three biological replicates were tested for each strain. Data were analysed using GraphPad Prism 5 and statistical significance assessed using the student’s t-test.

### Toxin A-specific and toxin B-specific Western Blots and Vero cell cytotoxicity assays

Toxins A and B were partially purified by methanol chloroform precipitation from culture supernatants harvested after 72 hours growth in TY broth, and detected by Western immunoblotting using specific antibodies as described previously [25]. Levels of cytotoxicity were tested in a Vero cell cytotoxicity assay using *C. difficile* culture supernatants harvested after 72 hours as described previously [24]. For the time course experiments, the same cultures as used for the sporulation assays described above were used. The assay endpoint (toxin titre) was recorded as the reciprocal of the last dilution in which full cytopathic effect (CPE) was observed. Three biological replicates were tested for each strain. Data were analysed using GraphPad Prism 5 and statistical significance assessed using the student’s t-test.

### Vero cell viability assays

Viability assays were performed as before [31] using the same tissue culture plates that were used for the Vero cell cytotoxicity assays described above. Following the scoring of CPE, a 20 µl aliquot of thiazolyl blue tetrazolium bromide (MTT; Sigma) at a concentration of 0.5% (w/v) in PBS was added to each well and the plate shaken gently. The plates were then incubated for 4 h at 37°C in 5% CO_2_ in the dark. The MTT-containing culture medium was then removed from each well and 200 µl of dimethyl sulfoxide (Sigma) was added and the plate shaken gently to ensure all formazan dye had dissolved. Absorbance was then measured using a Tecan infinite M200 plate reader with a test wavelength of 570 nm and a reference wavelength of 630 nm. Data were analysed using GraphPad Prism 5 and statistical significance assessed using ANOVA.

### Growth and motility assays

*C. difficile* cultures were subcultured from –80°C glycerol stock onto HIS plates containing 1% (w/v) agar. Plates were incubated anaerobically at 37°C for up to four days. Colony morphology was analysed visually, and documented using an Olympus BX-51 microscope. In addition, strains were grown overnight in HIS broth and 15 µl aliquots then spotted onto HIS plates containing 1% (w/v) agar. The plates were incubated for up to four days to allow growth on the surface of the agar plates. Growth was analysed visually and documented.

Swimming motility was assayed using the method outlined in Reynolds *et al.* (2011)[32]. Overnight HI broth cultures were used to stab inoculate HI medium containing 0.175% (w/v) agar to a depth of 3 cm. Motility was also tested in TY medium. Selection was included in the overnight cultures but not in the motility media. Motility was analysed visually after incubating for 24 hours.

## Results

### Tn*916-*mediated conjugation facilitates efficient transfer of plasmid DNA from *B. subtilis* to the clostridia

Previously, our laboratory developed a Tn*916*-mediated conjugation system to facilitate the transfer of plasmid DNA from a *Clostridium perfringens* donor into *C. difficile* ribotype 027 strains [24]. Whilst this system proved efficient, the counter-selection step used to remove the *C. perfringens* donor was sub-optimal, with high background levels of the donor often encountered. These high background levels of *C. perfringens* made it difficult to isolate the desired *C. difficile* transconjugants, particularly if the plasmid was transferred only at low frequency (unpublished data). Thus, we sought an alternate donor and so extended this system to include the use of *Bacillus subtilis* BS34A which carries the broad host range conjugative transposon Tn*916* [33]. *B. subtilis* is sensitive to both D-cycloserine and cefoxitin, allowing the use of double counter-selection and we hypothesised that this antibiotic sensitivity, coupled with the poor growth of this species under anaerobic conditions [34], might restrict the level of background donor growth. The shuttle plasmid pDLL4 [24] was introduced into BS34A and plate matings using *C. difficile*, *C. perfringens*, *Clostridium septicum* and *Clostridium sordellii* recipient strains were performed. For comparative purposes, matings were also performed using *C. perfringens* JIR4225 as a donor strain, as had been previously performed [24].

As can be seen in Table 3, pDLL4 was successfully transferred from the *B. subtilis* donor to all recipient strains tested, indicating the broad utility of this system for plasmid transfer into the clostridia. The frequency of plasmid transfer into *C. difficile* recipient strains was estimated to be approximately 10-fold higher than those achieved when using *C. perfringens* JIR4225 as the donor strain. In addition, the background of *B. subtilis* donor cells was substantially less than the background of *C. perfringens* donor cells, making the isolation of *C. difficile* transconjugants much easier and less time consuming than before. Also of note is the transfer of recombinant plasmid DNA into *C. perfringens* strain JIR39. Although relatively few transconjugants were obtained, this strain has remained refractory to genetic manipulation using all other techniques for the past thirty years. This result further highlights the robustness of our optimized system for plasmid transfer into the clostridia.

**Table 3:** Efficiency of Tn*916*-mediated plasmid transfer to the clostridia from a *C. perfringens* JIR4225 or a *B. subtilis* BS34A donor.

Recipient strain	Organism	*C. perfringens* JIR4225 mediated plasmid transfer (transconjugants/ml)	*B. subtilis* BS34A mediated plasmid transfer (transconjugants/ml)
JIR8094	*C. difficile*	1×10^1^ – 6×10^3^	9.4×10^1^ – 2.5×10^4^
CD37	*C. difficile*	3×10^1^ – 5×10^3^	2×10^2^ – 4.2×10^4^
VPI10463	*C. difficile*	2.1×10^3^– 1.8×10^3^	1.6×10^2^ – 2.4×10^4^
M7404	*C. difficile*	5.6×10^1^ – 2.4×10^3^	2.3×10^2^ – 5×10^4^
CD196	*C. difficile*	2×10^1^ – 1.7×10^3^	8.8×10^2^ – 3.8×10^4^
JGS6133	*C. difficile*	6.3×10^1^– 2.1×10^3^	4.5×10^2^ – 4.3×10^4^
SM101	*C. perfringens*	1.5×10^4^ – 5×10^4^	6.1×10^3^ – 5×10^4^
JIR39	*C. perfringens*	5×10^1^ – 5×10^3^	1×10^1^ – 1.5×10^2^
ATCC9714	*C. sordellii*	1.5×10^3^ – 2×10^3^	1×10^3^ – 9×10^5^
BX96	*C. septicum*	5×10^1^ – 8×10^2^	1×10^1^ – 2.5×10^3^

### Construction of *spo0A* mutants in *C. difficile* using *B. subtilis* as a donor strain for plasmid transfer

**Figure 1:**
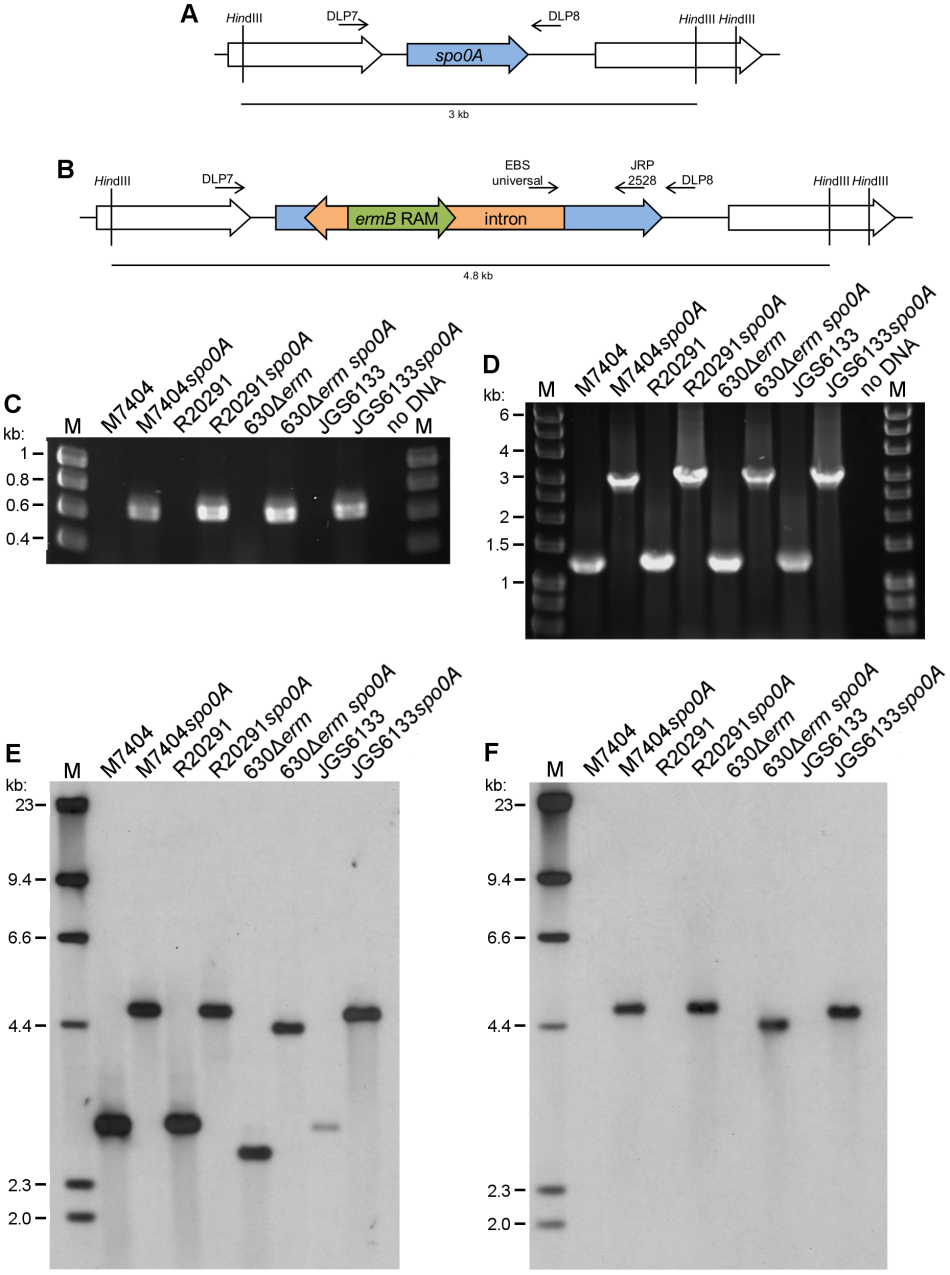
Confirmation of *spo0A* mutant construction in *C. difficile* strains by PCR and Southern blot. Diagram of *spo0A* and surrounding genes in the wild type strain R20291 (A) and mutant derivative in which the targetron has inserted (B), showing primers and restriction sites used to confirm disruption of *spo0A* in *C. difficile*. PCR using the EBS-universal primer and the *spo0A*-specific primer JRP2528 resulted in a product of approximately 0.6 kb for the mutant strains, while no product was generated for the wild type parental strains (C). PCR with primers flanking *spo0A* showed that the mutant generated a product of approximately 3 kb, confirming an insertion of approximately 1.8 kb was present in these strains when compared to wild type which produced a product of 1.2 kb (D). Southern blot analysis using probes specific for *spo0A* (E) and the intron (F) confirmed the disruption of *spo0A* with the targetron. The probe for *spo0A* hybridised to a band of 3 kb for the wild type strains M7404, R20291 and 630Δ*erm* and 2.7 kb for JGS6133, while for each mutant the probe hybridised to a band 1.8 kb greater. The intron-specific probe hybridised to a band of the corresponding size in the mutants only and did not bind to wild type strain DNA.

To test the new plasmid transfer system for use in mutagenesis studies, *spo0A* was chosen as a target due to the ease of screening for loss of the sporulation phenotype. The ribotype 027 strains M7404 and R20291 were used as recipients, as were the ribotype 012 derivative 630Δ*erm* and the ribotype 078 porcine strain JGS6133. Mutants were constructed as outlined in Materials and Methods. PCR using the targetron-specific EBS-universal primer (5’ CGAAATTAGAAACTTGCGTTCAGTAAAC 3′) and the *spo0A*-specific primer JRP2528 (5′ GATAATGTTGAGCTTTTAGGTGCAG 3′) and Southern hybridisation analysis were used to confirm disruption of *spo0A* (Figure 1). All *spo0A* mutants constructed were complemented with an intact copy of *spo0A* on a multi-copy plasmid, pDLL16. The sequence of *spo0A* and immediate upstream region is the same in R20291, M7404, 630 and JGS6133 (data not shown). For this reason, we used the same construct to complement the *spo0A* mutation in each strain background. The vector pDLL4 was also introduced into the wild type parent strains and mutant derivatives in order to maintain the same antibiotic selection across all of the strains during phenotypic assays. Note that to ensure that any phenotypic differences observed between the wild type and *spo0A* mutant derivatives were not the result of altered growth kinetics, growth curve experiments were performed. The growth of the *spo0A* mutants *in vitro* over a 12 hour period was not significantly different to wild type in each of the strain backgrounds tested (Figure S1) which is in agreement with other studies which also found that disruption of *spo0A* in *C. difficile* does not affect growth rates *in vitro* [13], [14]. In addition, cellular morphology appeared to be unaffected in all of the *spo0A* mutants and complemented derivatives. Gram staining and light microscopy showed that the wild type, mutant and complemented derivatives of each panel of strains were not discernably different under the conditions tested (Figure S2A-D), consistent with previous studies [13], [14].

**Figure 2:**
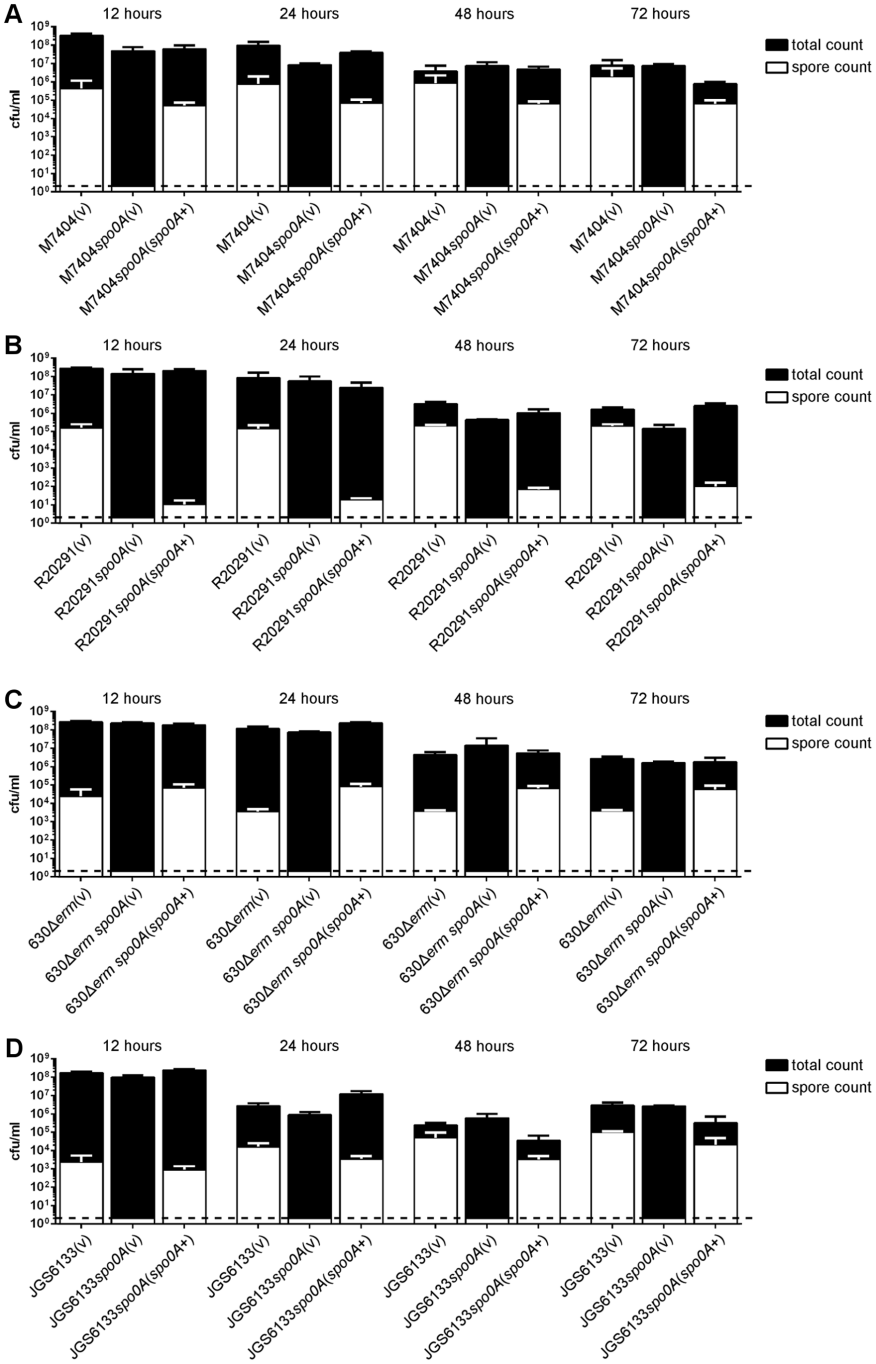
Characterisation of sporulation over time in each of the *spo0A* mutants. The wild type, mutant and complemented derivatives in the M7404 (A), R20291 (B), 630Δ*erm* (C) and JGS6133 (D) backgrounds were assayed for total viable count (black bars) and heat-shocked spore count (white bars) after incubation for 12, 24, 48 and 72 hours. Three biological replicates were tested for each strain; the mean values of these replicates are shown together with the standard errors of the means. The dashed line represents the limit of detection for this assay.

To confirm phenotypically that Spo0A function had been abolished and subsequently restored upon complementation, sporulation assays were performed on the isogenic panels of strains. The requirement of Spo0A for sporulation in *C. difficile* is already well established [13], [14], [29]. As expected, these assays showed that heat-resistant spores could not be detected in the mutant strains, while heat-resistant spores were observed for the wild type strains and complemented derivatives (Figure 2A-D). Complementation of *spo0A* in both the M7404 and JGS6133 mutants resulted in wild-type levels of sporulation (p > 0.05) (Figure 2A and 2D). When the 630Δ*erm spo0A* mutant was complemented, the levels of sporulation were the same as or higher than the wild type (Figure 2C). By contrast, complementation of the R20291 *spo0A* mutant was not as successful in this assay. While heat-resistant spores were observed, the levels produced by the complemented strain were significantly lower than wild-type at each time point tested (p  =  0.0001 – 0.0377) (Figure 2B).

### Spo0A represses toxin A and toxin B production in *C. difficile* ribotype 027 strains

**Figure 3:**
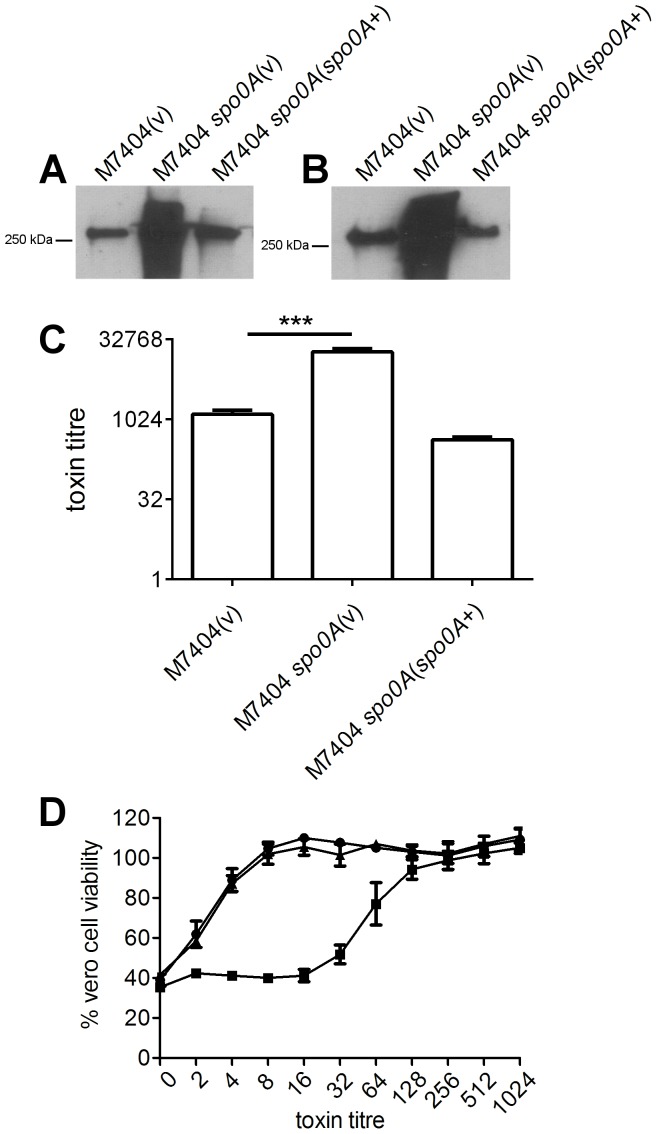
Analysis of toxin A and toxin B production by M7404 *spo0A* mutant. The wild type, mutant and complemented derivatives were examined for toxin production by Western immunoblotting using (A) TcdA-specific and (B) TcdB-specific antibodies and precipitated supernatant proteins from the strains indicated. Toxin production by these strains was also quantified. Serial doubling dilutions of culture supernatants were made in MEM alpha medium supplemented with 1% HI FCS and used in Vero cell cytotoxicity assays (C). Morphological changes of the Vero cells were observed and scored by microscopy after 24 hrs. The toxin titre is the reciprocal of the endpoint dilution. Vero cell viability (D) was also measured using MTT reagent. •, M7404 (vector control); ▪, M7404 *spo0A* mutant (vector control); ▴, complemented derivative. All assays were performed in duplicate on at least three independent culture supernatants; the mean values of these assays are shown together with the standard errors of the means. ***, p ≤ 0.001. For Western immunoblot analysis, three independent culture supernatants were tested; the image shown is representative.

**Figure 4:**
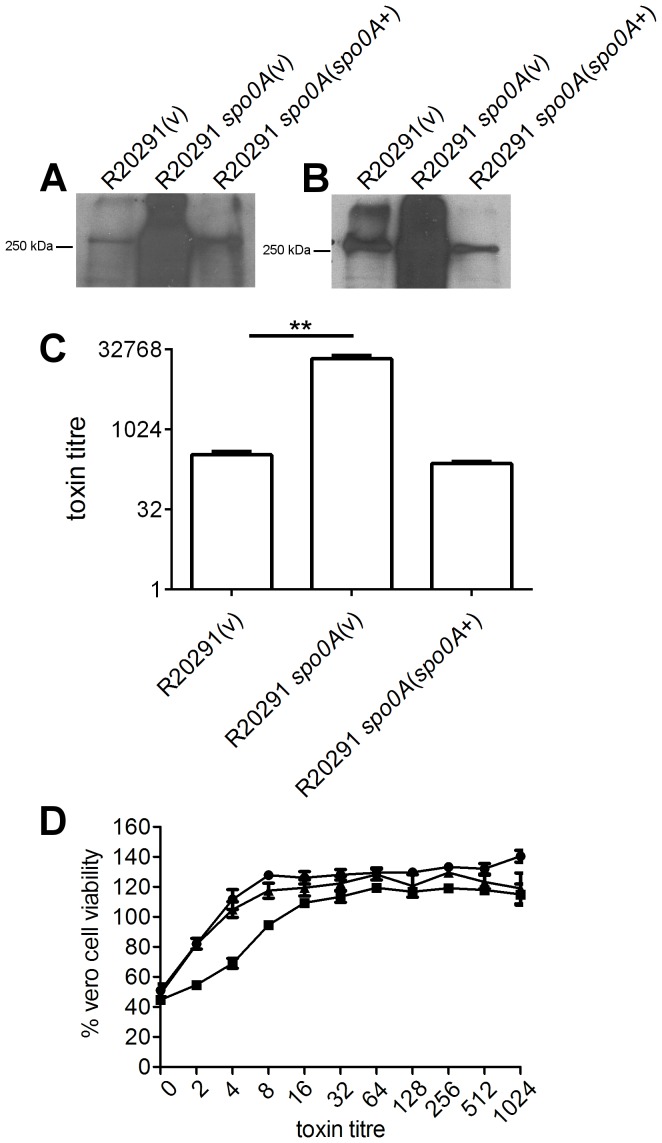
Analysis of toxin A and toxin B production by R20291 *spo0A* mutant. The wild type, mutant and complemented derivatives were examined for toxin production by Western immunoblotting using (A) TcdA-specific and (B) TcdB-specific antibodies and precipitated supernatant proteins from the strains indicated. Toxin production by these strains was also quantified. Serial doubling dilutions of culture supernatants were made in MEM alpha medium supplemented with 1% HI FCS and used in Vero cell cytotoxicity assays (C). Morphological changes of the Vero cells were observed and scored by microscopy after 24 hrs. The toxin titre is the reciprocal of the endpoint dilution. Vero cell viability (D) was also measured using MTT reagent. •, R20291 (vector control); ▪, R20291 *spo0A* mutant (vector control); ▴, complemented derivative. All assays were performed in duplicate on at least three independent culture supernatants; the mean values of these assays are shown together with the standard errors of the means. **, p ≤ 0.01. For Western immunoblot analysis, three independent culture supernatants were tested; the image shown is representative.

To initially assess the role of Spo0A in toxin A and toxin B production by the ribotype 027 strains M7404 and R20291, Western immunoblot analyses using toxin-specific antibodies were performed using culture supernatants harvested after 72 hours growth. The M7404 *spo0A* mutant appeared to produce substantially more toxin A (Figure 3A) and toxin B (Figure 3B) than wild type. The same observation was made with the R20291 *spo0A* mutant, with increased levels of toxin A and toxin B seen compared to wild type (Figure 4A and 4B). Importantly, the levels of both toxins appeared to be restored to approximately wild type levels in the complemented derivatives (Figures 3 and 4).

To confirm this result, and to assess whether the observed increase of toxin in the ribotype 027 *spo0A* mutants resulted in a difference in activity compared to wild type, doubling dilution cytotoxicity assays were performed on Vero cells, which are particularly sensitive to the effects of toxin B. These assays confirmed the results of the Western immunoblot analysis. Toxin-containing supernatant from the M7404 *spo0A* mutant showed a statistically significant (p  =  0.001) increase in the amount of cytotoxic activity compared to the wild type parental strain, which was restored to wild type levels upon complementation (Figure 3C). The R20291 *spo0A* mutant demonstrated the same trend, with the *spo0A* mutant in this background also producing a statistically higher (p  =  0.0015) amount of cytotoxic activity compared to wild type (Figure 4C). Complementation restored these levels to wild type. Vero cell viability was also measured using an MTT assay. The M7404 *spo0A* mutant showed a statistically significant decrease in Vero cell viability compared to wild type (p  =  0.0037) (Figure 3D) as did the R20291 mutant (p  =  0.0003) (Figure 4D). In each of the ribotype 027 isolates, complementation restored Vero cell viability to the same levels as wild type. Taken together, these data suggest that Spo0A negatively regulates toxin production in ribotype 027 strains of *C. difficile*.

### Spo0A does not affect toxin production in the *C. difficile* ribotype 012 derivative 630Δ*erm*

**Figure 5:**
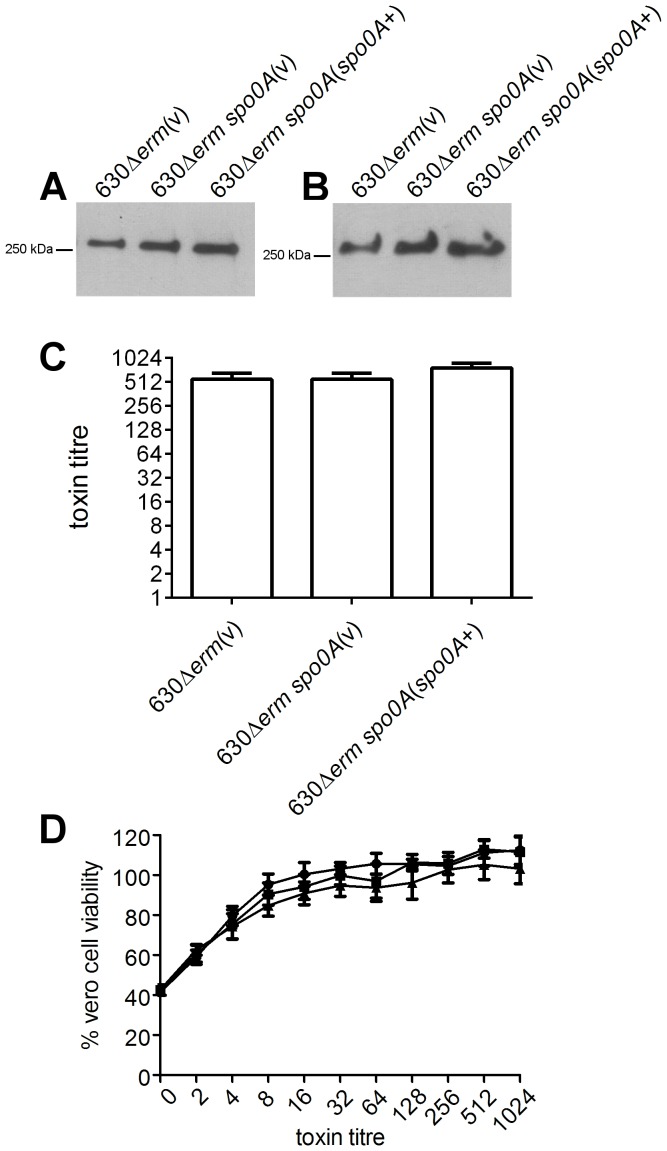
Analysis of toxin A and toxin B production by 630Δ*erm spo0A* mutant. The wild type, mutant and complemented derivatives were examined for toxin production by Western immunoblotting using (A) TcdA-specific and (B) TcdB-specific antibodies and precipitated supernatant proteins from the strains indicated. Toxin production by these strains was also quantified. Serial doubling dilutions of culture supernatants were made in MEM alpha medium supplemented with 1% HI FCS and used in Vero cell cytotoxicity assays (C). Morphological changes of the Vero cells were observed and scored by microscopy after 24 hrs. The toxin titre is the reciprocal of the endpoint dilution. Vero cell viability (D) was also measured using MTT reagent. •, 630▵*erm* (vector control); ▪, 630▵*erm spo0A* mutant (vector control); ▴, complemented derivative. All assays were performed in duplicate on at least three independent culture supernatants; the mean values of these assays are shown together with the standard errors of the means. For Western immunoblot analysis, three independent culture supernatants were tested; the image shown is representative.

Western immunoblot analysis was also performed on the 630Δ*erm* isogenic panel of strains, to assess the role of Spo0A on toxin A and toxin B production in this strain background. By contrast to the findings with the ribotype 027 strains, disruption of Spo0A in 630Δ*erm* does not appear to affect toxin A or toxin B production. There was little qualitative difference in the levels of toxin A and B in the supernatants collected from the wild type 630Δ*erm* strain, the *spo0A* mutant and complemented derivative (Figure 5A and 5B). In addition, there was no significant difference in the levels of cytotoxicity (Figure 5C) or Vero cell viability (Figure 5D) suggesting that under the conditions tested, Spo0A does not regulate toxin production in this strain.

### Spo0A does not affect toxin production during later growth phases in the *C. difficile* ribotype 078 strain JGS6133

**Figure 6:**
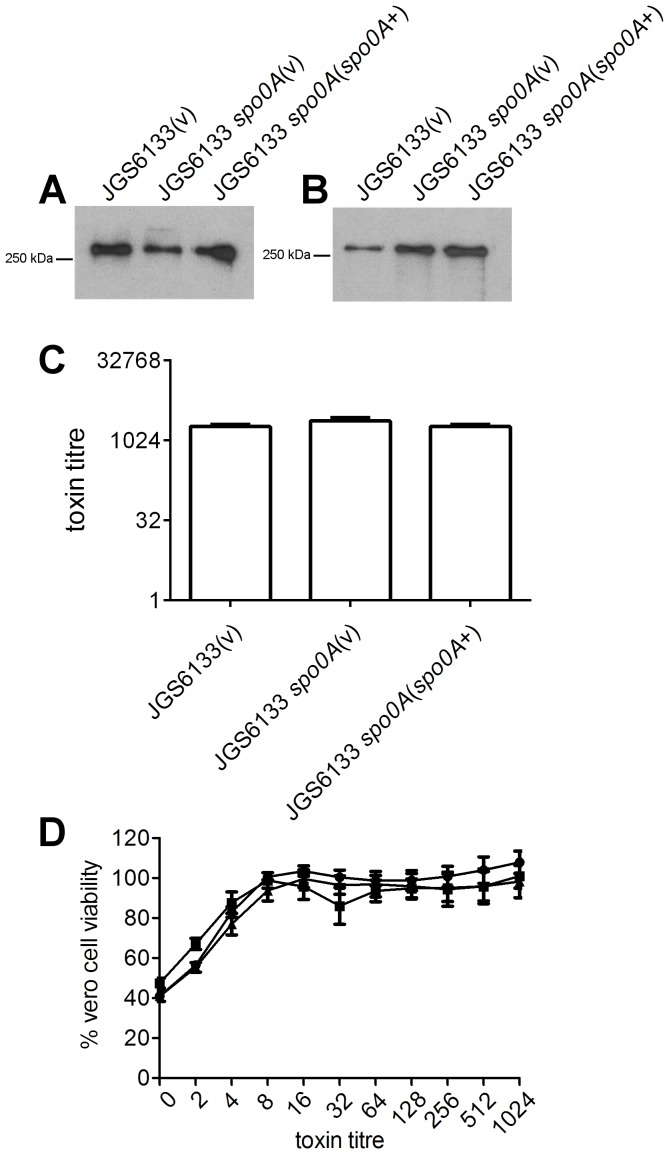
Analysis of toxin A and toxin B production by JGS6133 *spo0A* mutant. The wild type, mutant and complemented derivatives were examined for toxin production by Western immunoblotting using (A) TcdA-specific and (B) TcdB-specific antibodies and precipitated supernatant proteins from the strains indicated. Toxin production by these strains was also quantified. Serial doubling dilutions of culture supernatants were made in MEM alpha medium supplemented with 1% HI FCS and used in Vero cell cytotoxicity assays (C). Morphological changes of the Vero cells were observed and scored by microscopy after 24 hrs. The toxin titre is the reciprocal of the endpoint dilution. Vero cell viability (D) was also measured using MTT reagent. •, JGS6133 (vector control); ▪, JGS6133 *spo0A* mutant (vector control); ▴, complemented derivative. All assays were performed in duplicate on at least three independent culture supernatants; the mean values of these assays are shown together with the standard errors of the means. For Western immunoblot analysis, three independent culture supernatants were tested; the image shown is representative.

Since Spo0A appeared to regulate toxin production in the two ribotype 027 strains tested but not in the ribotype 012 derivative 630Δ*erm*, we decided to construct an additional *spo0A* mutant in a third and divergent *C. difficile* genetic background, namely the ribotype 078 strain JGS6133. Again, Western immunoblot analysis was performed to initially assess the role of Spo0A on toxin A and toxin B production using culture supernatants harvested after 72 hours. As with 630Δ*erm*, little qualitative difference in the levels of toxin A and toxin B could be observed between the JGS6133 wild type, *spo0A* mutant and complemented derivative at this time point (Figure 6A and 6B). This was confirmed by the Vero cell cytotoxicity assays (Figure 6C) and Vero cell viability (Figure 6D), both of which showed no significant difference between the wild type, *spo0A* mutant, and complemented strains. Collectively, these results indicate that Spo0A is involved in the negative regulation of toxin A and toxin B in the ribotype 027 isolates M7404 and R20291. However, under these conditions, Spo0A does not appear to be involved in the regulation of these two major toxins in either the ribotype 012 derivative 630Δ*erm* or the ribotype 078 isolate JGS6133.

### Spo0A differentially regulates toxin production over time in the ribotype 078 strain but not the ribotype 027 isolates tested

**Figure 7:**
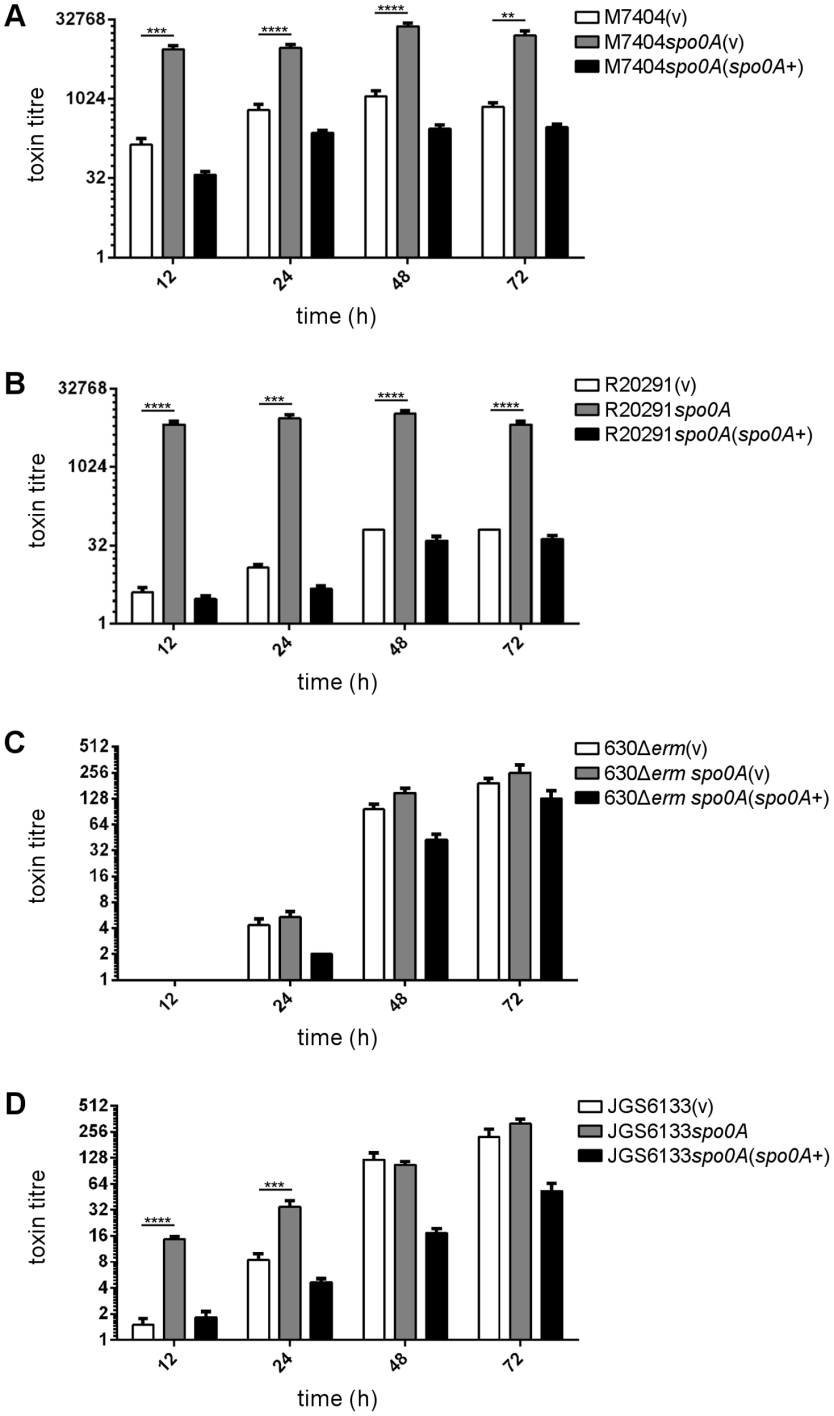
Time course analysis of cytotoxic activity of *C. difficile spo0A* mutants. The wild type, mutant and complemented derivatives of M7404 (A), R20291 (B), 630Δ*erm* (C), and JGS6133 (D) were examined for toxin production over 12, 24, 48 and 72 hours. Serial doubling dilutions of culture supernatants were made in MEM alpha medium supplemented with 1% HI FCS and used in Vero cell cytotoxicity assays. Morphological changes of the Vero cells were observed and scored by microscopy after 24 hrs. The toxin titre is the reciprocal of the endpoint dilution. All assays were performed in duplicate on at least three independent culture supernatants; the mean values of these assays are shown together with the standard errors of the means. **, p ≤ 0.01; ***, p ≤ 0.001; ****, p ≤ 0.0001.

The above experiments examining toxin production focused on this phenotype after 72 hours of growth. To determine if Spo0A differentially regulated toxin production in *C. difficile* at earlier time points, a time course experiment was performed. In this assay, samples of culture supernatant were taken after 12, 24, 48, and 72 hours of growth and toxin activity ascertained using the Vero cell cytotoxicity assay (Figure 7A-D). As before, no statistically significant difference was observed between wild type and *spo0A* mutant in the strain 630Δ*erm* background, regardless of the time point tested (Figure 7C). The JGS6133 *spo0A* mutant again was not significantly different to the wild type at 72 hours, a trend which was also observed at 48 hours (Figure 7D). Interestingly, however, the JGS6133 *spo0A* mutant showed higher levels of cytotoxicity compared to wild type at both 12 hours (p < 0.0001) and 24 hours (p  =  0.0007). Complementation restored cytotoxicity to levels the same as, or lower than, wild type at each time point tested (Figure 7D). For both of the ribotype 027 strains tested, the *spo0A* mutant showed markedly greater levels of cytotoxicity at each time point tested (p ≤ 0.0001 – 0.0016) (Figure 7A and 7B). The level of cytotoxicity observed at 12 hours and 72 hours was not significantly different for either the M7404 *spo0A* mutant or R20291 *spo0A* mutant. Again, complementation restored cytotoxicity to levels the same as, or lower than, wild type at each time point tested (Figure 7C and 7D). These data suggest that Spo0A differentially regulates toxin production in these strain backgrounds, with temporal changes in regulation in the ribotype 078 strain but not in the two ribotype 027 isolates tested. Thus, the regulatory pathways controlled or influenced by Spo0A in relation to toxin production in these various strain backgrounds clearly differ.

### Spo0A does not appear to be involved in flagella-mediated motility

**Figure 8:**
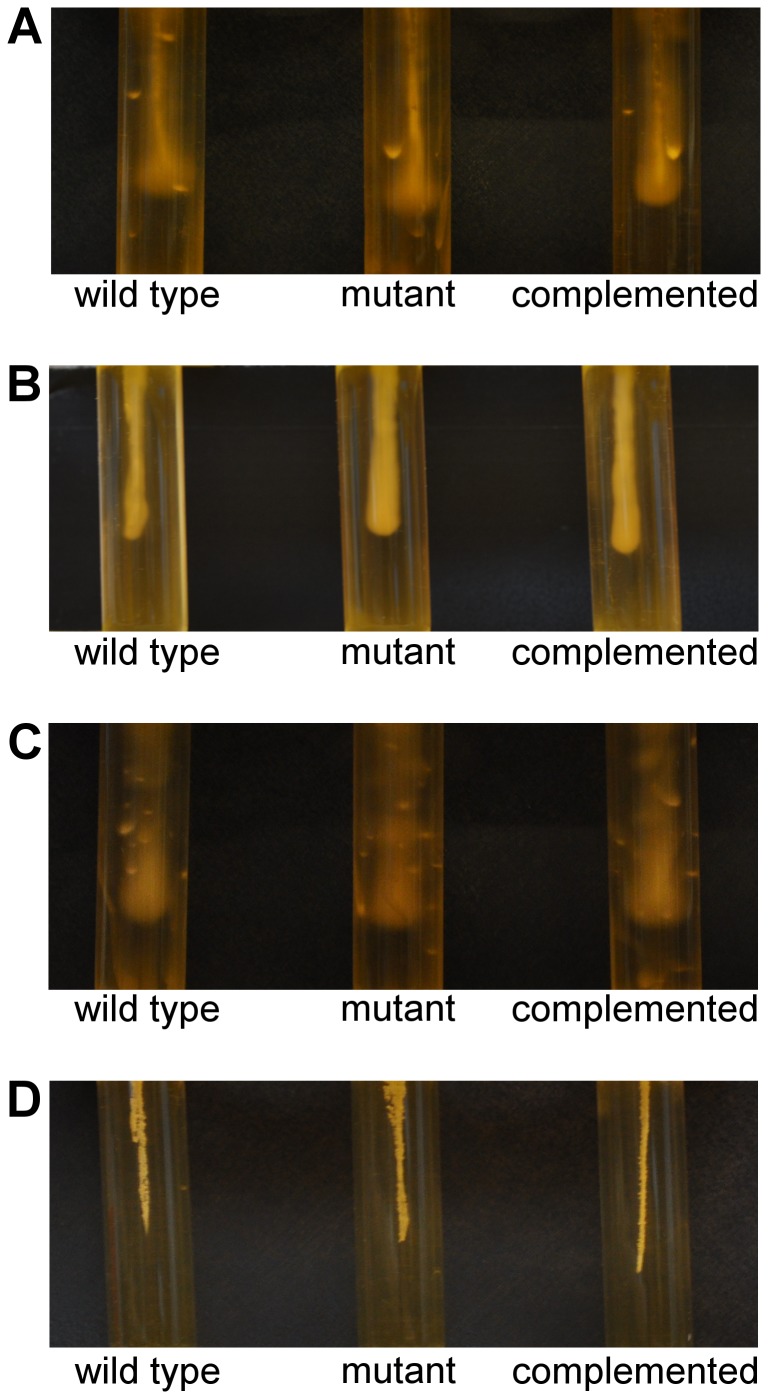
Disruption of *spo0A* in *C. difficile* does not affect *in vitro* swimming motility. Overnight cultures of the isogenic panels of wild type, mutant and complemented derivatives were used to inoculate semi-solid (0.0175% (w/v)) HIS agar. After 24 hours incubation, motility was observed for the M7404 derivatives (A), the R20291 derivatives (B) and the 630▵*erm* derivatives (C); JGS6133 and derivatives are non-motile under these conditions (D). No clear observable difference was noted between wild type and *spo0A* mutant for any strain background tested. The assay was performed in technical duplicates and repeated three times for each strain. Images are representative of each assay.

In *Bacillus* spp. part of the network controlled by Spo0A is flagella-mediated swimming motility. To test whether Spo0A is involved in the control of swimming motility in *C. difficile*, overnight cultures of the wild type, mutant and complemented strains were used to inoculate semi-solid (0.175% (w/v)) HI agar. After 24 hours incubation, motility was observed and recorded. The mutant derivatives of M7404, R20291 and 630Δ*erm* show no observable difference in the ability to swim in semi-solid HIS medium compared to wild type (Figure 8A-C). JGS6133 does not display swimming motility (Figure 8D). Swimming motility was also tested using the more nutritionally limited TY medium, with no difference observed (data not shown). Under the conditions tested here, there does not appear to be a link between Spo0A and swimming motility in *C. difficile*.

### Disruption of *spo0A* in *C. difficile* results in an altered colony morphology

**Figure 9:**
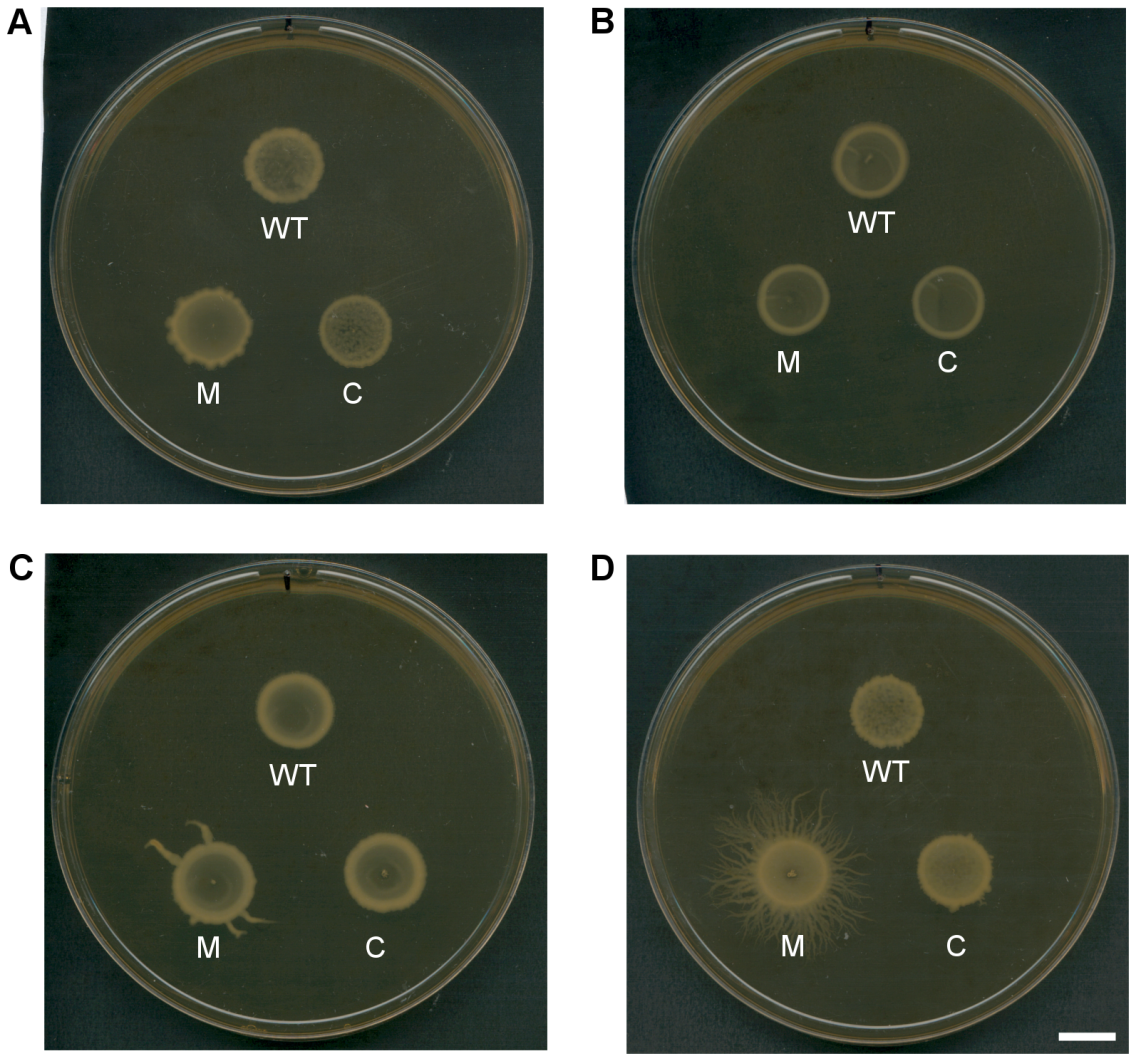
Disruption of *spo0A* in *C. difficile* leads to altered motility on solid medium. For each strain a 15 µl spot of overnight culture was placed onto a 1% (w/v) HIS agar plate which was then incubated for four days. Following incubation, the plates were observed for any changes in growth across the agar surface in the mutant compared to wild type and the complemented derivative for strains M7404, R20291, 630▵*erm* and JGS6133 (A-D, respectively). WT  =  wild type; M  =  *spo0A* mutant; C  =  complemented. The white scale bar represents 1 cm. Images are representative of three independent experiments.

To examine bacterial growth on an agar plate surface, broth cultures of each strain were grown overnight and 15 µl aliquots were spotted onto 1% (w/v) HIS agar. After incubation for four days, growth was visually recorded (Figure 9A-D). The R20291 *spo0A* mutant looked indistinguishable from wild type (Figure 9B), however, the M7404 *spo0A* mutant appeared to have more ruffled edges than its wild type parent (Figure 9A). The 630Δ*erm spo0A* mutant produced “flare”-like growth, while the complemented derivative appeared more like wild type (Figure 9C). Most distinct was the JGS6133 *spo0A* mutant which produced many branching tendrils from the centre spot of growth, which were not present in the wild type and were lost upon complementation (Figure 9D).

**Figure 10:**
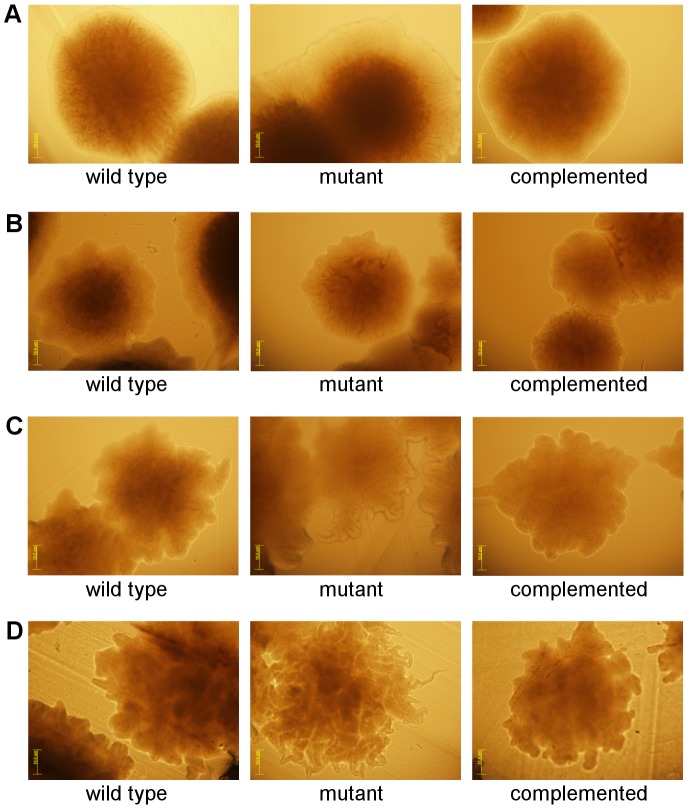
Colony morphology is altered in *C. difficile spo0A* mutants. For each strain a 10 µl aliquot of glycerol stock was spread across a 1% (w/v) HIS agar plate. After incubation, colonies were examined microscopically for any morphological changes in the mutant compared to wild type and the complemented derivatives for strains M7404, R20291, 630▵*erm* and JGS6133 (A-D, respectively). The yellow scale bar in each image is 10 µm. Images are representative of three independent experiments.

To examine colony morphology microscopically, a 10 µl aliquot of the glycerol stock of each strain was spread across a 1% (w/v) HIS agar plate. After incubation, colonies were examined for any changes in morphology (Figure 10A-D). The colony edges of M7404 looked smooth, as did the complemented *spo0A* mutant (Figure 10A). The M7404 *spo0A* mutant had a more ruffled edge in comparison to the wild type, with a more pronounced halo-like edge surrounding the colony centre (Figure 10A). The other ribotype 027 isolate, R20291, displayed a slightly more ruffled colony edge compared to M7404, however, the R20291 wild type, *spo0A* mutant and complemented derivative strains were not markedly different to each other (Figure 10B). Both 630Δ*erm* and JGS6133 wild type demonstrated more irregular edges than either M7404 or R20291 wild type (Figure 10C and 10D). The 630Δ*erm spo0A* mutant showed a tendril-like morphology in comparison to the wild type while the complemented derivative was not observably different to wild type (Figure 10C). The JGS6133 *spo0A* mutant showed a more hairlike and finer morphology compared to the defined boundary of the wild type and complemented strains (Figure 10D). These results suggest that Spo0A may be involved in the regulation of growth or motility of *C. difficile* across a solid surface.

## Discussion

Improved methods for the introduction of plasmids into *C. difficile* recipient strains have broadened the range of strains which can now be examined. The laboratory-derived strain 630Δ*erm* has been widely used as a model for *C. difficile* pathogenesis studies due to the relative ease with which it can be manipulated in the laboratory and the availability of the 630 genome sequence [35], the first *C. difficile* genome sequence to be published. More recently, the focus has moved to ribotype 027 isolates after their emergence a decade ago as a serious clinical issue, and developing an understanding of their spread and “hypervirulence” became imperative. Now, other strain backgrounds such as ribotype 078 are becoming more prevalent and have been associated with more severe disease [18], in addition to their role in the emergence of veterinary infections [21], [36], [37]. Part of this study involved the expansion of our developing genetic techniques into other strain backgrounds, through the use of a *B. subtilis* donor and Tn*916*-mediated plasmid transfer. We coupled this system with targetron technology in order to construct *spo0A* mutants in a range of strain backgrounds, with the first mutant constructed in a ribotype 078 background described here. The isolate that we chose to use, JGS6133, is a porcine ribotype 078 isolate from the United States. Ribotype 078 isolates are one of the most common strain types identified in pig populations globally [21], [36] but are also increasingly found in the human population [18], [38], making this a particularly relevant strain type to study.

As expected, we found that Spo0A plays an important role in sporulation in each strain tested since the *spo0A* mutants displayed an asporogenous phenotype which could be restored upon complementation. Complementation of the sporulation phenotype was not complete in all strain backgrounds, with the R20291 complemented derivative producing fewer heat-resistant spores than its wild type parent. This contrasts with the other ribotype 027 isolate tested, M7404, in which the sporulation phenotype was completely restored. It also contrasts with the toxin assays in which full complementation of toxin activity was observed for both M7404 and R20291. Few studies have attempted to complement a *spo0A* mutation in *C. difficile*; those which have complemented this mutation demonstrate varying degrees of success. Deakin *et al.* (2012) tested isogenic panels of 630Δ*erm* and R20291 strains and found *in vitro* levels of sporulation were restored to wild type levels in their complemented derivatives [14]. When they tested the R20291 strains for toxin production, however, the complemented strain still produced increased levels of toxin compared to wild type (indeed, the opposite of what we see here with our R20291 panel of strains). Dawson *et al*. (2012) found that sporulation levels (when tested in a biofilm model) were not restored to wild type in their complemented R20291 *spo0A* mutant [39]. They also suggested that Spo0A may be involved in protection against oxygen stress, finding the *spo0A* mutant was more susceptible to oxygen stress than wild type. However, again, the phenotype was not fully restored in the complemented strain [39]. Each of these studies, and our own, introduced an intact copy of *spo0A* on a multicopy plasmid into the relevant mutants to achieve complementation. The lack of consistency in complementing various phenotypes suggests this method is not optimal for complementation of the range of regulatory networks Spo0A may be connected to.

In addition to sporulation, we assessed the role of Spo0A in toxin A and toxin B production in the ribotype 027, 012 and 078 strain backgrounds. We found that Spo0A represses toxin A and toxin B production in ribotype 027 isolates of *C. difficile* as upon disruption of *spo0A* in the ribotype 027 isolates R20291 and M7404 a clear increase in the production of both toxin A and toxin B was observed; as discussed above, complementation restored these levels to approximately those displayed by the wild type. In support of these findings, Deakin *et al.* (2012) observed that a R20291 *spo0A* mutant caused more severe disease in a murine model than the wild type strain, and associated this increase in severity with an increase in the amount of toxin A and toxin B produced by the mutant *in vitro*. This study also looked at strain 630Δ*erm* and found neither the wild type nor the *spo0A* derivative produced overt disease in the murine model [14]. Unfortunately, this study did not report cytotoxicity data for 630Δ*erm* or its *spo0A* mutant [14]. Thus, while the 630Δ*erm spo0A* mutant does not appear phenotypically different in the *in vivo* model tested in this study, we cannot make any comparisons about *in vitro* toxin production between this work and our own.

Our finding that disruption of *spo0A* does not affect major toxin production in 630Δ*erm* contrasts with that of Underwood *et al*. (2009) who have suggested that Spo0A positively regulates toxin A and toxin B production in this strain [13]. Possibly, experimental variation could account for the differences observed. This previous study tested toxin production with strains grown in BHI medium which contains glucose, a known repressor of toxin production in *C. difficile* [40], while TY was used in our study to avoid any confounding effects of glucose. However, a more recent study also tested 630Δ*erm* and a *spo0A* mutant, finding no statistically significant difference between wild type and mutant regardless of whether TY or BHIS media were used [12]. This later study is supported by our finding that Spo0A does not regulate toxin production in 630Δ*erm*. Unfortunately, neither study [12], [13] tested a complemented derivative or independent mutant, so it is not possible to draw any conclusions about secondary mutations in their strains. The varied findings of these two studies, in addition to our own, highlight the need to test complemented strains – or, if complementation cannot be achieved, an independent mutant – to confirm the results observed.

How Spo0A is able to influence toxin production in ribotype 027 and 078 strains is unknown. It is also not clear why there is a temporal difference in regulation of toxin production in the ribotype 078 strain tested, but not in the two ribotype 027 strains tested. Transcriptomic analysis, while deemed outside the scope of this initial study, would perhaps elucidate the role of Spo0A in these different strain backgrounds. In *B. subtilis* levels of active (phosphorylated) Spo0A influence the bacterium’s transition from one phenotype to another [41]. We do not yet know if this is the case in *C. difficile*. Active (phosphorylated) Spo0A can act directly on a target gene by binding upstream at consensus 0A boxes, or can act indirectly by influencing the transcription of target genes that then themselves result in the up- or down-regulation of other genes [42]. It has been suggested that Spo0A is able to bind upstream of *tcdB in vitro* [12], however, no 0A boxes have been located upstream of any PaLoc genes [13]. One study reports that 0A boxes have been identified at approximately 100 sites in the 630 genome [12], while another notes that putative 0A boxes have been identified upstream of more than 200 open reading frames [13]. Both of these figures suggest that Spo0A is potentially involved in the direct regulation of a number of phenotypes. In *B. subtilis*, Spo0A has been associated with the regulation of over 500 genes including those involved in motility [8]. For this reason, we examined whether Spo0A influenced motility by *C. difficile*. No change was observed in swimming motility by the *spo0A* mutants constructed in M7404, R20291 or 630Δ*erm* under the conditions used in this study. As expected, no swimming motility was observed for JGS6133; isolates of toxinotype V, to which JGS6133 belongs, have been shown to lack components required for biosynthesis of the flagella apparatus [43]. By contrast, genes encoding the type IV pili biosynthesis appear to be part of the core gene set and are conserved across diverse strains of *C. difficile* [43], [44]. Thus, we also examined whether Spo0A influenced the growth of *C. difficile* on a solid agar surface. The altered appearance of colony growth on the surface of 1% (w/v) agar plates may suggest a role for Spo0A in surface motility such as twitching motility, which is mediated by type IV pili [45], [46], or gliding or sliding movement which occurs independently of type IV pili [46]. Again, a strain-specific phenomenon was observed with the JGS6133 *spo0A* mutant demonstrating a markedly distinct change in growth on solid agar plates. In contrast, the changes seen with the M7404 and 630Δ*erm* mutants were less drastic in comparison to wild type, while the R20291 mutant did not display any observable changes compared to wild type. However, the mechanistic change responsible for the altered colony morphology observed is currently unknown and requires further investigation.

The regulation of cell behaviours in *C. difficile* is clearly complex and will rely on a number of different regulatory systems which may vary among strain types. How these various regulatory systems are linked to Spo0A is currently unknown, as is how Spo0A influences such a range of different phenotypes in *C. difficile*. It appears that toxin production and sporulation are not directly associated, as the *spo0A* mutants are unable to sporulate but still produce toxins A and B [13], [14] as is also the case for a *sigH* mutant [47]. Thus, Spo0A likely regulates these phenotypes through other mediators which may be otherwise unconnected to each other. It is possible that these mediators are different in the various strain types of *C. difficile*. *C. difficile* is a genetically diverse species and shows low genome conservation, with a core gene set estimated at approximately 20% [43], [44], [48]. Five clonal lineages have been identified [22], [23] and there are genomic differences between these clades which may result in phenotypic differences related to important parameters such as disease severity or persistence. It is already well established that the ribotype 027 strains are different to other strains such as 630. Comparative genome analysis of ribotype 027 strains and isolate 630 revealed a number of putative ribotype 027-specific regulators, with eight additional two component systems identified as well as 15 transcriptional regulators [49]. It is possible that these ribotype 027-associated regulatory systems could be influencing toxin production *via* Spo0A. Targeted disruption of these regulators, coupled with transcriptional and phenotypic analyses, could help elucidate the role they play and whether they interact with Spo0A. The ribotype 078 strains appear genetically divergent from other *C. difficile* types [22], [23] and further comparative analysis of the regulatory networks in this strain background is required.

The study reported here highlights the functional differences which may be seen in the representative strain types tested. The development of genetic techniques in *C. difficile*, in our laboratory and in others, has enabled the greater exploration of potential virulence factors in this organism. It is becoming increasingly clear that the regulation of various phenotypes in *C. difficile* is extremely complex and varies across evolutionarily diverse strains. This complexity may be compounded by genetic changes occurring due to the continual passage of laboratory strains over the last twenty to thirty years. The loss of erythromycin resistance in strain 630▵*erm*, while crucial to the development of genetic systems, was the result of continual strain passage and will have resulted in other, unknown, spontaneous mutations. Some of these may affect toxin production. When tested in our laboratory, 630▵*erm* displays higher levels of cytotoxicity than the wild type 630 strain or an independently derived erythromycin sensitive derivative, JIR8094 [27], suggesting a loss of negative regulation in this derivative (data not shown). Perhaps this loss of negative regulation in 630Δ*erm* is obscuring any effect that disrupting Spo0A is having on toxin production. Given the results of this study, construction of regulation mutants in wild type 630 would be beneficial to elucidate the role of these proteins in the original strain background. However, another reason for the differences seen in our study could be natural variation across strain backgrounds, perhaps relating to niche adaptation. The ribotype 078 strain used in this study, JGS6133, has not undergone constant passaging in our laboratory, unlike 630Δ*erm*. As noted above, ribotype 078 strains appear to be genetically divergent from other *C. difficile* strains [22], [23] and may have adapted to fill a different environmental space than other strain types such as ribotype 027 or 012. It is thus reasonable to expect there will also be differences between this strain type and others. How we can untangle which mechanism is responsible for the differences we observe when conducting classical mutagenesis studies is part of the future challenge for *C. difficile* genetic studies. The data presented in this study further highlight the heterogeneous nature of *C. difficile*, and demonstrate the continued need to study not only historical isolates, but also new and emerging strains of this important pathogen.

## Supporting Information

Figure S1 **Growth kinetics are unaffected in** ***C. difficile spo0A*** **mutants.** The OD_600_ for each strain was measured every hour for 12 hours to determine any change in the mutant compared to wild type and the complemented derivatives for strains M7404, R20291, 630▵*erm* and JGS6133 (A-D, respectively). The growth of the *spo0A* mutants *in vitro* over a 12 hour period was not significantly different to wild type in each of the strain backgrounds tested. Growth was measured for at least three independent culture supernatants for each strain; the mean values of these assays are shown together with the standard errors of the means. Statistical significance was assessed using ANOVA.
(TIF)

Figure S2 **Gross cellular morphology is unaffected in** ***C. difficile spo0A*** **mutants.** Each strain was grown on a HIS agar plate containing thiamphenicol selection for 48 hours. Gram stains were performed and slides examined under brightfield microscopy for any morphological changes in the mutant compared to wild type and the complemented derivatives for strains M7404, R20291, 630▵*erm* and JGS6133 (A-D, respectively).
(TIF)
