# Supplementary figures and images for: Spo0A Differentially Regulates Toxin Production in Evolutionarily Diverse Strains of Clostridium difficile
Source: PLoS One. 2013 Nov 13;8(11):e79666. doi: 10.1371/journal.pone.0079666 (PMC3827441; doi:10.1371/journal.pone.0079666)

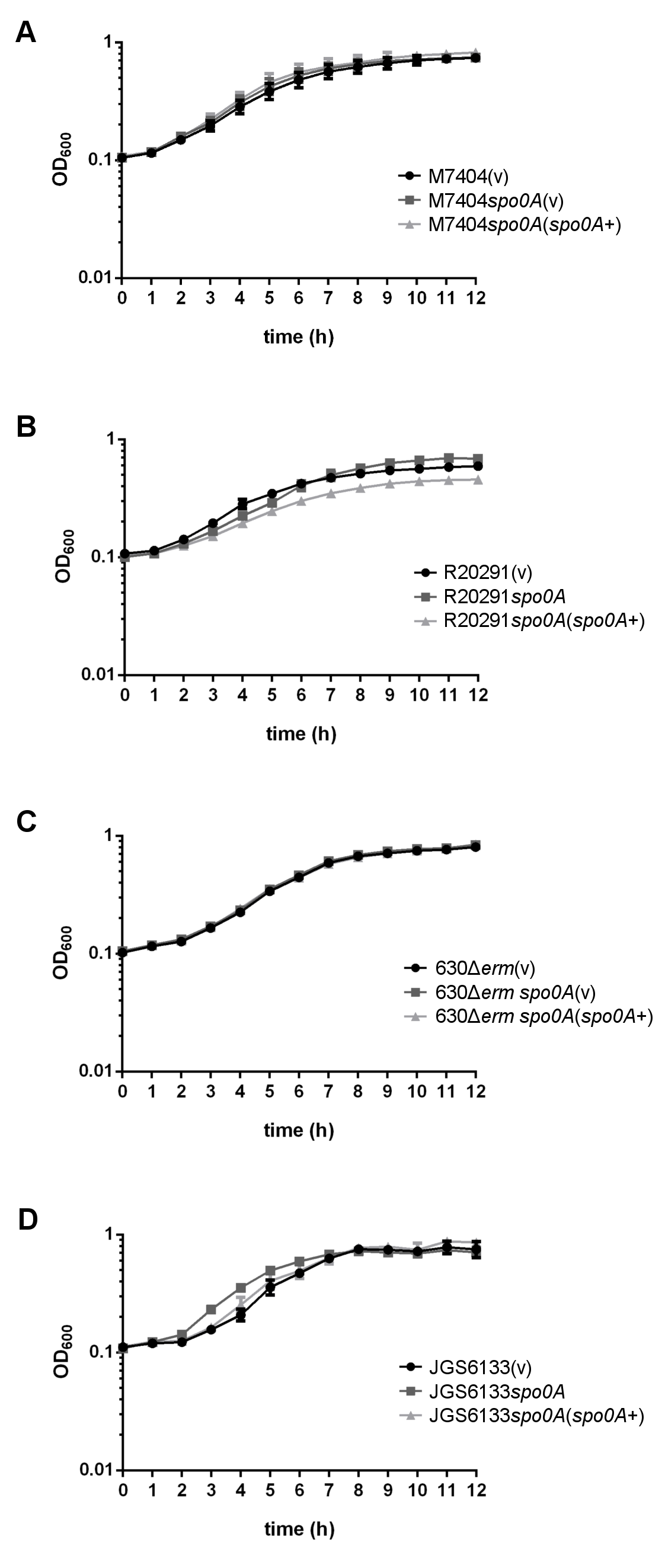

Supplement: Figure S1 — Growth kinetics are unaffected in C. difficile spo0A mutants. The OD600 for each strain was measured every hour for 12 hours to determine any change in the mutant compared to wild type and the complemented derivatives for strains M7404, R20291, 630▵erm and JGS6133 (A-D, respectively). The growth of the spo0A mutants in vitro over a 12 hour period was not significantly different to wild type in each of the strain backgrounds tested. Growth was measured for at least three independent culture supernatants for each strain; the mean values of these assays are shown together with the standard errors of the means. Statistical significance was assessed using ANOVA. (TIF) [file pone.0079666.s001.tif]

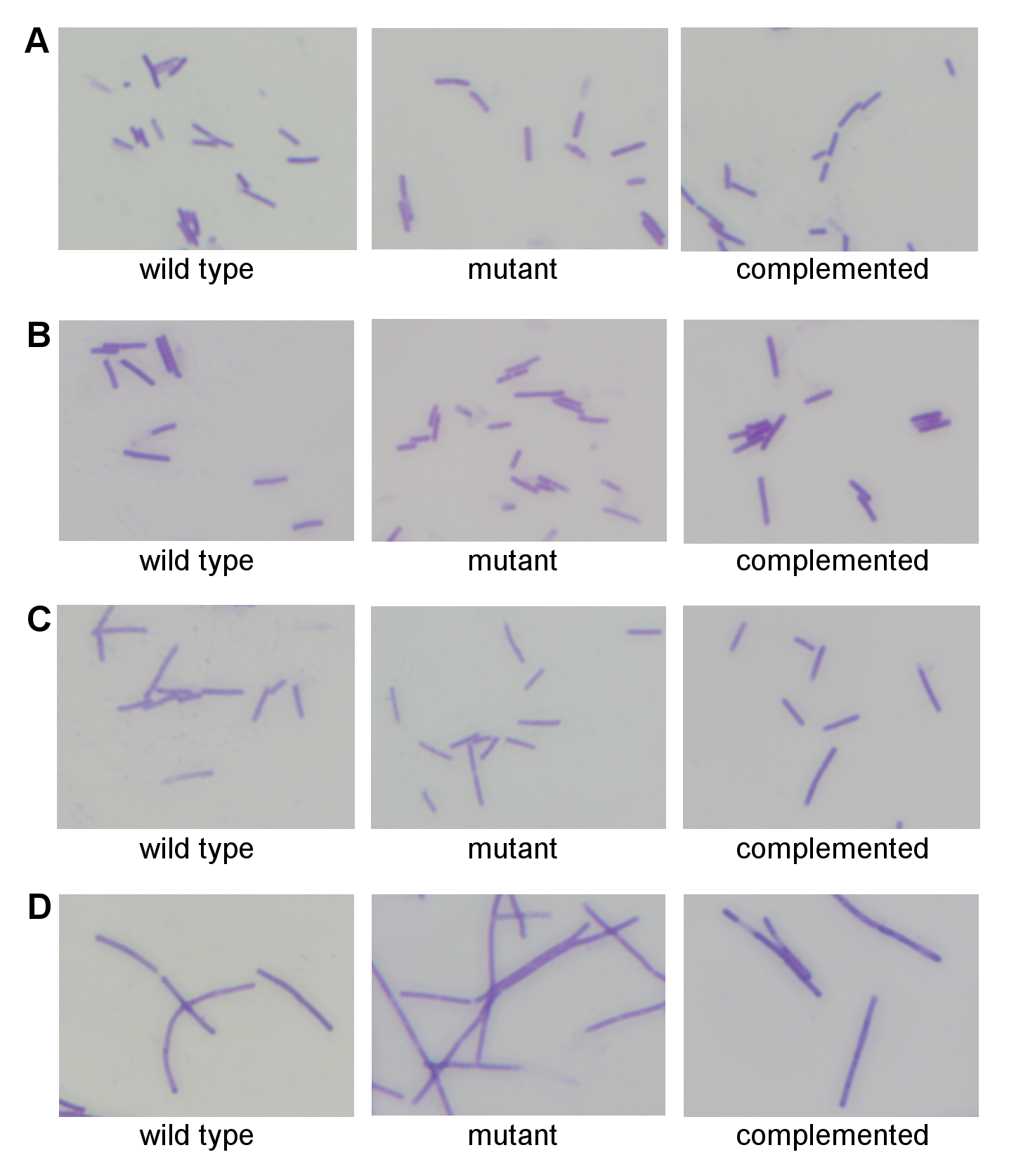

Supplement: Figure S2 — Gross cellular morphology is unaffected in C. difficile spo0A mutants. Each strain was grown on a HIS agar plate containing thiamphenicol selection for 48 hours. Gram stains were performed and slides examined under brightfield microscopy for any morphological changes in the mutant compared to wild type and the complemented derivatives for strains M7404, R20291, 630▵erm and JGS6133 (A-D, respectively). (TIF) [file pone.0079666.s002.tif]
